# 

**Published:** 1867-10

**Authors:** 


### CONTENTS.

### ORIGINAL CONTRIBUTIONS.

ART. XLII. On the Cause and Treatment of Panniform Corneæ, Occurring with Granular Ophthalmia. By Jos. S. Hildreth, M.D., Chicago, ---- 577

ART. XLIII. Report on Cholera in 1866. By H. Wardner, M.D., Chairman of Committee, ---- 582

ART. XLIV. Vis Conservatrix. By F. R. Payne, M.D., ---- 589

ART. XLV. Cases of Intermittent and Remittent Fever, Treated with Chloroform, Internally. By Geo. F. Brickett, M.D., of Chicago, ---- 594

ART. XLVI. Interesting Case of Suspension of Growth of a Fœtus in Utero, at about the Seventh Month of Gestation—Its subsequent Death and Delivery at Full Term By [illegible] D. Mercer, of Omaha, Neb., ---- 597

### FOREIGN CORRESPONDENCE.

Letter from Edmund Andrews, M.D., ---- 599

" " " " " On Venereal Diseases and Prostitution under the License System of Europe.—The Laws, Statistics, and Results of the System, ---- 603

### SELECTIONS.

Contributions to Practical Surgery. By John Mettauer, M.D., LL.D., Prince Edward C.H., Virginia, ---- 616

### BOOK NOTICES.

Chemistry. By William Thomas Brande, D.C.L., F.R.S.L., and Alfred Swaine Taylor, M.D., F.R.S., ---- 622

A Treatise on Human Physiology. By John C. Dalton, M.D., ---- 623

A Treatise on Emotional Disorders of the Sympathetic System of Nerves. By Williams Murray, M.D., M.R.C.P., ---- 623

Clinical Lectures on the Principles and Practice of Medicine. By John Hughes Bennett, M.D., F.R.S.E., ---- 624

The Physician's Visiting List for 1868, ---- 624

Injuries of the Eye, Orbit, and Eyelids; Their Immediate and Remote Effects. By George Lawson, F.R.C.S., ---- 624

### EDITORIAL.

Chicago Medical College, ---- 625

Rush Medical College, ---- 625

Chicago Medical Society, ---- 626

Public Health, ---- 626

Medical Books, ---- 626

Apology, ---- 626

Chicago Charitable Eye and Ear Clinic, ---- 626

To Medical Colleges, ---- 627

Prizes offered by the Conn. Medical Society for 1868, ---- 630

New Mode of Treating Fracture of Lower Jaw, ---- 631

Sulphite of Soda in the Treatment of Erysipelas, ---- 632

Phthisis in Barbadoes, ---- 633

French Students, ---- 633

Mortality Report for the Month of August, ---- 634

Sloughing Produced by Local Anæsthesia, ---- 635

Money Receipts, ---- 636

To Physicians, ---- 636

Statistics of Cholera in Italy, ---- 636

Curare in Epilepsy, ---- 636

